# Compact High-Zoom-Ratio Mid-Wavelength Infrared Zoom Lens Design Based on Particle Swarm Optimization

**DOI:** 10.3390/s25020467

**Published:** 2025-01-15

**Authors:** Zhenhao Liu, Jipeng Zhang, Yuqi Huang, Xin Zhang, Hongbo Wu, Jianping Zhang

**Affiliations:** 1 Changchun Institute of Optics, Fine Mechanics and Physics, Chinese Academy of Sciences, Changchun 130033, China; liuzhenhao18@mails.ucas.ac.cn (Z.L.); zhangjipeng20@mails.ucas.ac.cn (J.Z.); huangyuqi20@mails.ucas.ac.cn (Y.H.); optlab@ciomp.ac.cn (X.Z.); 2 University of Chinese Academy of Sciences, Beijing 100049, China; 3 State Key Laboratory of Applied Optics, Changchun 130033, China; 4 Key Laboratory of Optical System Advanced Manufacturing Technology, Chinese Academy of Sciences, Changchun 130033, China

**Keywords:** zoom lens design, high zoom ratio, particle swarm optimization, optimization algorithms

## Abstract

This paper presents an automated method for solving the initial structure of compact, high-zoom-ratio mid-wave infrared (MWIR) zoom lenses. Using differential analysis, the focal length variation process of zoom lenses under paraxial conditions is investigated, and a model for the focal power distribution and relative motion of three movable lens groups is established. The particle swarm optimization (PSO) algorithm is introduced into the zooming process analysis, and a program is developed in MATLAB to solve for the initial structure. This algorithm integrates physical constraints from lens analysis and evaluates candidate solutions based on key design parameters, such as total lens length, zoom ratio, Petzval field curvature, and focal length at tele end. The results demonstrate that the proposed method can efficiently and accurately determine the initial structure of compact MWIR zoom lenses. Using this method, a mid-wave infrared zoom lens with a zoom ratio of 50×, a total length of less than 530 mm, and the ratio of focal length to total length approximately 2:1 was successfully designed. The design validates the effectiveness and practicality of this method in solving the initial structure of zoom lenses that meet complex design requirements.

## 1. Introduction

Zoom lenses play a crucial role in modern optical systems, enabling simultaneous wide-field observation and high-resolution focusing capabilities [1,2]. Over the years, advancements in zoom lens design have been driven by increasing demands from industries such as photography, surveillance, and machine vision. For example, in reconnaissance missions, portable zoom lenses can be used for long-range observation or reconnaissance, especially in aerial reconnaissance or drone applications. Compact lenses with a large zoom ratio offer high flexibility, accommodating different observation distances and target sizes. In particular, mid-wavelength infrared (MWIR) systems, used extensively in thermal imaging and remote sensing, require compact designs with high zoom ratios to meet performance and spatial constraints.

There are three main methods for determining the initial structure of a zoom lens. The first uses the paraxial wavefront curvature (PWC) approach to calculate lens parameters [3], offering precise results but becoming increasingly complex with higher zoom ratios and more lens elements. The second references existing patent literature; however, limited data on compact, high-zoom-ratio lenses reduces its practicality [4]. The third method analyzes the zoom process, determining motion trajectories and focal power distributions under near-axis conditions, and refining the structure using optical design software. This approach accurately describes the zoom process and avoids discontinuities, but is complex, requiring preset parameters, iterative adjustments, and extensive experience. Additionally, as the initial structure is a multi-solution problem, evaluating solution quality is crucial for effective design [5,6,7,8]. To address these challenges, this paper proposes combining computer optimization algorithms with differential equation of zoom lens analysis [9]. Iterative parameter adjustments and clear evaluation criteria improve solution quality, providing a solid foundation for further design.

Optimization algorithms such as genetic algorithms, ant colony optimization, simulated annealing, neural networks, and particle swarm optimization (PSO) are widely used in optical design. Among these, PSO is simple to implement, requiring only particle initialization and parameter adjustments, unlike the complex operations in genetic algorithms and simulated annealing. It converges quickly, especially in early optimization, outperforming genetic algorithms and simulated annealing in speed. PSO adapts well to complex, high-dimensional, and non-differentiable problems with lower computational costs than genetic algorithms. Its global search capability balances local and global optimization, maintaining efficiency and robustness. Compared to genetic algorithms and simulated annealing, PSO has fewer parameter tuning difficulties and avoids common pitfalls like local optima traps. These make it particularly suitable for optical system optimization. Hua Qin was the first to apply PSO to the spherical aberration correction of a single aspherical lens [10]; Zichao Fan conducted theoretical derivation for the fixed foci zoom section of a dual-telecentric zoom lens, and used PSO to achieve a zoom ratio of 7× [11,12]; XIAO YU et al. applied PSO to achieve a zoom ratio of 12.4× for a dual-conjugate zoom lens in a fast steering mirror system [13], and since their research focuses on a specific structure, the requirements for this special structure impose additional constraints on the movement laws of the lens groups within the system. This exclusion of certain solutions during the solving process makes it difficult to achieve a larger zoom ratio. Chengxiang Fan et al. combined Gaussian bracket methods with PSO to design a zoom lens with a zoom ratio of 20×, using a focal tunable lens as an additional compensating group [14]. The disadvantage of this design lies in the need for additional mechanical components to achieve the focal length adjustment of the focusing lens, which is unfavorable for the miniaturization of the lens. Moreover, since the medium is liquid, the system becomes more sensitive to temperature changes, affecting the performance of the system. This characteristic also limits the potential application scenarios.

Building on these advancements, this study presents an automated approach for determining the initial structure of a compact, high zoom ratio, MWIR zoom lens. By integrating PSO with paraxial optical analysis, the proposed method addresses design challenges associated with physical constraints, multi-solution problems, and computational efficiency. Unlike traditional trial-and-error methods, this approach offers a systematic framework to efficiently evaluate candidate solutions within a predefined search space.

This paper is organized as follows: Section 2 introduces the theoretical analysis of zoom lens systems and the PSO algorithm. Section 3 elaborates on the solution process, while Section 4 provides a design and verification example for a MWIR zoom lens. Section 5 discusses the proposed method and proposes subsequent improvements. Section 6 concludes the study.

## 2. Theories

### 2.1. Theoretical Analysis of Zoom Lens Systems

A zoom lens typically consists of multiple fixed and movable lens groups. The movable groups include a zoom group, which adjusts the focal length, and a compensation group, responsible for correcting defocus. Compensation can be achieved through optical or mechanical methods, with the latter being more suitable for compact and continuous zoom systems.

In two-movable-lens-group designs, increasing the zoom ratio often leads to challenges such as extreme focal power distribution and aberrations or larger system sizes due to increased displacement of lens groups. To mitigate these issues, this study introduces a third movable lens group to the conventional two-group system. By redistributing the zoom ratio among the lens groups, the design achieves a shorter zoom stroke while maintaining the desired zoom ratio [15].

**Figure 1:**
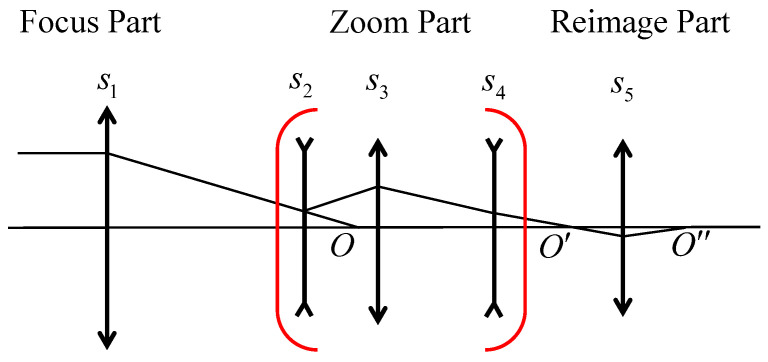
Paraxial structure diagram of a secondary imaging optical system.

Figure 1 illustrates the paraxial structure of a mid-wave infrared (MWIR) zoom lens. The system adopts a secondary imaging configuration and consists of a front fixed lens group S1, three movable lens groups S2, S3, and S4, and a relay group S5.

The first group, S1, has a positive focal power and is designed to collect incoming light. The second group, S2, serves as the zoom group, while the third S3 and fourth S4 groups function as compensation groups. S2, with negative focal power, adjusts the light collected by S1, reducing both the incidence angle on the image plane and the height of incident light on S3. To further converge the light from the first two groups and reduce the overall system size, S3 is designed with positive focal power. Theoretically, when the first three groups are configured as “+−+”, they can form a reverse telephoto structure at long focal configuration, which can provide a focal length that is much longer than the total length of the system.

After determining the focal powers of the first three groups, there are two possible configurations for the focal power of the fourth group: “+−+−” and “+−++” [5]. Based on theoretical analysis and experimental results, it is observed that if a zoom lens system contains only one negative focal power group, the focal length of this negative power group will be relatively short. Consequently, the curvature of the lenses in this group will be very large, leading to increased aberrations. Therefore, a configuration with two negative focal power groups “+−+−” is selected, as it allows for a more balanced distribution of focal power across the lens groups.

**Figure 2:**
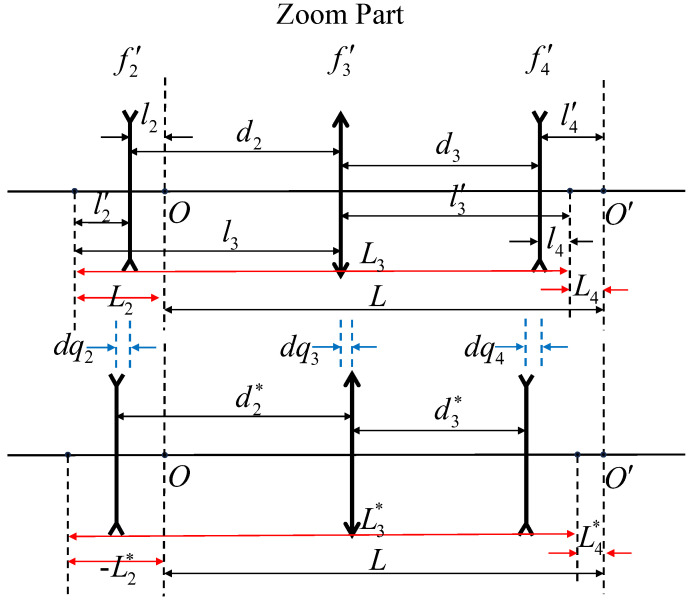
Diagram of movable lens groups in the zoom lens system.

Before calculating the specific focal power of each lens group, an analysis of the zooming process is necessary. Figure 2 depicts the zooming mechanism of the lens.

This figure illustrates the arrangement and movement of the lens groups within the zoom lens system. It highlights the roles of each movable lens group (S2, S3, and S4) during the zooming process, showing their relative positions and travel paths as the system adjusts focal length. *L* represents conjugated distance, and $dq_{i}$ represents the slight displacement of each movable lens group.

As the focal length of the zoom lens changes, the lens group S2 undergoes displacement. This displacement causes a shift in the image plane *O*. To compensate for this shift and maintain the stability of the imaging plane $O^{\prime }$, the other two movable groups, S3 and S4, also move accordingly. This ensures that the conjugate distance between the object and image planes within the zooming section remains constant. Finding this compensation relationship over the entire focal length range of the zoom lens essentially involves solving the paraxial structure of the system.

Firstly, the conjugate distances $L_{i}$ for each lens group in the system can be expressed as follows:

(1) $$L_{i}=f_{i}\left(2-\frac{1}{\beta_{i}}-\beta_{i}\right)$$

where *i* represents the lens groups in the zoom part, i=2,3,4. $f_{i}$ and $\beta_{i}$ representing the focal length and magnification of the lens group in the zoom part.

In the initial state, the conjugate distance of the system can be expressed as

(2) $$L\left(\beta_{2},\beta_{3},\beta_{4}\right)=-f_{2}^{\prime }\left(2-\frac{1}{\beta_{2}}-\beta_{2}\right)+f_{3}^{\prime }\left(2-\frac{1}{\beta_{3}}-\beta_{3}\right)+f_{4}^{\prime }\left(2-\frac{1}{\beta_{4}}-\beta_{4}\right)$$

where the magnification *m* of the zoom part can be expressed as $m=\beta_{2}\beta_{3}\beta_{4}$, and the focal length of zoom lens without relay group can be expressed as $F=f_{1}\beta_{2}\beta_{3}\beta_{4}$.

When the focal length of the system changes after the lens groups in the system move, the conjugate distance of the system can be expressed as

(3) $$L\left(\beta_{2},\beta_{3},\beta_{4}\right)=-f_{2}^{\prime }\left(2-\frac{1}{\beta_{2}^{*}}-\beta_{2}^{*}\right)+f_{3}^{\prime }\left(2-\frac{1}{\beta_{3}^{*}}-\beta_{3}^{*}\right)+f_{4}^{\prime }\left(2-\frac{1}{\beta_{4}^{*}}-\beta_{4}^{*}\right)$$

the magnification $m^{*}$ of the zoom part can be expressed as $m^{*}=\beta_{2}^{*}\beta_{3}^{*}\beta_{4}^{*}$, and the focal length of zoom lens without relay group can be expressed as $F^{*}=f_{1}\beta_{2}^{*}\beta_{3}^{*}\beta_{4}^{*}$. Then, we can obtain the zoom ratio of the system by $R=m^{*}/m$ or $R=F^{*}/F$.

Since the conjugate distance of the system remains constant before and after the focal length change, the subtraction of the two equations can be obtained,

(4) $$-f_{2}^{\prime }\left(\frac{1}{\beta_{2}^{*}}+\beta_{2}^{*}-\frac{1}{\beta_{2}}-\beta_{2}\right)+f_{3}^{\prime }\left(\frac{1}{\beta_{3}^{*}}+\beta_{3}^{*}-\frac{1}{\beta_{3}}-\beta_{3}\right)+f_{4}^{\prime }\left(\frac{1}{\beta_{4}^{*}}+\beta_{4}^{*}-\frac{1}{\beta_{4}}-\beta_{4}\right)=0$$

From this equation, we can obtain a one-dimensional quadratic equation about the magnification of the compensation group,

(5) $$\beta_{3}^{*2}-b\beta_{3}^{*}+1=0$$

(6) $$b=\left(\frac{1}{\beta_{3}}+\beta_{3}\right)+\frac{f_{2}^{\prime }}{f_{3}^{\prime }}\left(\frac{1}{\beta_{2}^{*}}+\beta_{2}^{*}-\frac{1}{\beta_{2}}-\beta_{2}\right)-\frac{f_{4}^{\prime }}{f_{3}^{\prime }}\left(\frac{1}{\beta_{4}^{*}}+\beta_{4}^{*}-\frac{1}{\beta_{4}}-\beta_{4}\right)$$

Since the movement trajectory of the lens group needs to be coherent throughout the focal length change range of the zoom lens, it is necessary to maintain Equation (5) in the solution region, i.e., b<−2 or b>2 is required, where b>2 is the form of negative group compensation, so b<−2 is used as a constraint in this design [9].

The magnification of S2 and S4 has the following relationship with the amount of movement:

(7) $$\beta_{2}^{*}=\frac{1}{\frac{1}{\beta_{2}}-\frac{dq_{2}}{f_{2}^{\prime }}},\beta_{4}^{*}=\beta_{4}+\frac{dq_{4}}{f_{4}^{\prime }}$$

Substituting it into *b* gives the magnification of S3. Finally, the compensated displacement of S3 is obtained by the following equation:

(8) $$dq_{3}=f_{3}^{\prime }\left(\frac{1}{\beta_{3}^{*}}-\frac{1}{\beta_{3}}\right)-dq_{2}+f_{2}^{\prime }\left(\beta_{2}^{*}-\beta_{2}\right)$$

Based on the process described in Equations (1)–(8), we can use the given $f_{1},f_{2},f_{3}$ and $l_{2},d_{23},d_{34}$ to describe the zoom process of the zoom lens, and obtain the position of each lens group inside the zoom part when the zoom lens is at different focal lengths. The conjugate distance length *L* of the zoom part can also be obtained, as well as the maximum zoom ratio *R* that the system can provide by $R=m_{max}/m_{min}$.

In the actual solution process of the zoom length system, especially when the zoom ratio of the system increases significantly and the number of moving lens groups increases, in order to ensure that the image plane can be fully compensated at any time during the zoom process, and to maintain the continuity and smoothness of the zoom curve, it is necessary to carry out repeated parameter adjustment and calculation. This process is extremely time-consuming. Based on this, this study proposes a method for solving the initial structure of a high-zoom ratio zoom system based on particle swarm optimization algorithm.

### 2.2. Particle Swarm Optimization (PSO) Algorithm

The PSO algorithm, introduced by Kennedy and Eberhart, is a global optimization strategy inspired by the foraging behavior of bird flocks. The fundamental principle of PSO is to emulate the process by which individuals within a swarm communicate information to collectively find the optimal solution. Within a predefined search space, the movement of particles represents the exploration of candidate solutions to the objective function. The performance of each particle’s current position is evaluated using a fitness function, allowing the algorithm to record the particle’s historical best position (personal best). Simultaneously, through the exchange of information among particles, the algorithm identifies the swarm’s global best position [16].

So, the PSO algorithm has several core parameters: the number *N* of particles $X_{i}$ that make up a population $X=[X_{1},X_{2},\cdots ,X_{n}]$, the position $x_{i}$ of the particle $X_{i}$ within the search domain, the speed $v_{i}$ at which it moves within the search domain, and the individual optimal value Pbest. At the same time, a group optimal value Gbest can be determined by comparing individuals in the group. The swarm size *N* plays a critical role in the algorithm’s performance. If the swarm size is too small, the algorithm may lack sufficient diversity to escape local optima. Conversely, if the swarm size is too large, the improvement in optimization capability becomes marginal, and excessive computational resources are wasted. Therefore, selecting an appropriate swarm size is crucial for balancing solution quality and computational efficiency.

From the above, each particle has three basic characteristics.

1. Particle position: $x_{i}=\left(x_{1},x_{2},\ldots ,x_{D}\right)$ represents a candidate solution in the search space, *D* is the dimension of the particle;
2. Particle velocity: $v_{i}=\left(v_{1},v_{2},\ldots ,v_{D}\right)$ determines the particle’s movement within the search space;
3. Personal best: $Pbest_{i}$, the best position found by each particle individually.

**Figure 3:**
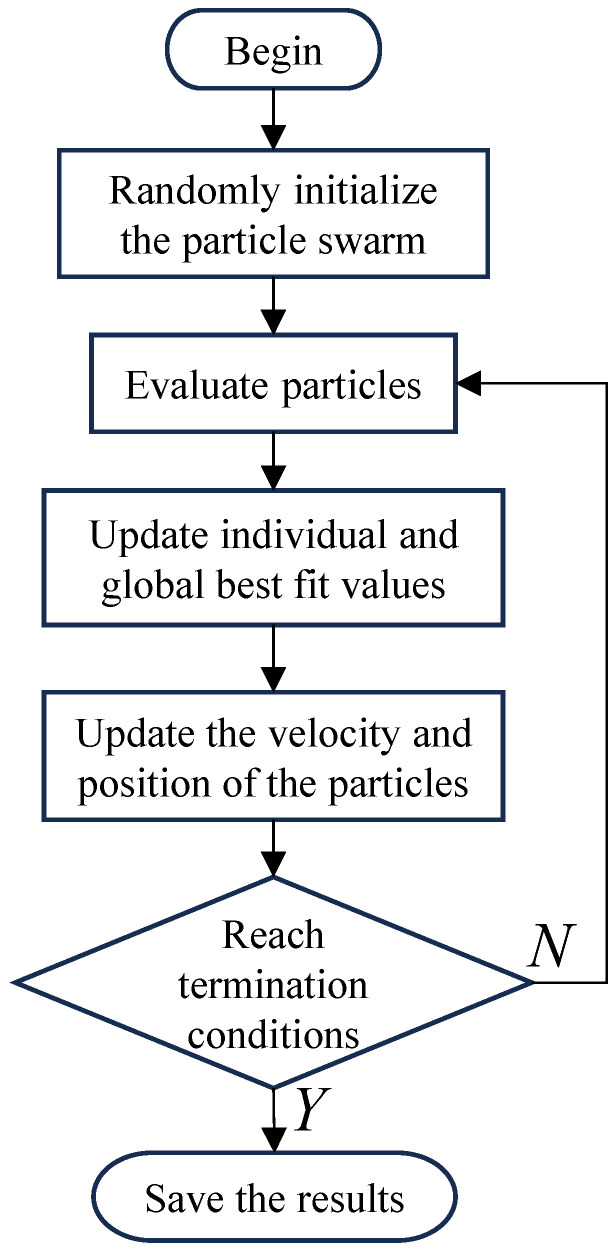
A flowchart of the zoom lens’ initial structure optimization based on particle swarm optimization algorithm.

The optimization process of the PSO algorithm is shown in Figure 3. At the beginning, we first randomly initialize the position *x* and velocity *v* of the particle in the search domain $S^{D}$. After evaluation of the evaluation function, an optimal value is recorded. The position and velocity of the particle are then updated in the next iteration by

(9) $$x_{i}^{k}=x_{i}^{k-1}+v_{i}^{k-1}$$

(10) $$v_{i}^{k}=w\cdot v_{i}^{k-1}+c_{1}r_{1}\left(Pbest_{i}-x_{i}^{k-1}\right)+c_{2}r_{2}\left(Gbest-x_{i}^{k-1}\right)$$

The meaning of Equation (9) is that the particle will move from its own position $x_{i}^{k-1}$ in the last iteration with the speed $v_{i}^{k-1}$ to $x_{i}^{k}$, where *k* is the current iteration number. The meaning of Equation (10) is that the velocity $v_{i}^{k}$ of the particle $x_{i}$ in the *k*th iteration is composed of three parts: the first part is the velocity $v_{i}^{k-1}$ of the particle $x_{i}$ itself in the last iteration; the second part is the best position $Pbest^{k}$ of the individual $x_{i}$ recorded by the particle; the third part is the best position $Gbest^{k}$ of the swarm *X* recorded by the group, and this step is the information exchange process among the particles in the group, in which the inertia coefficient *w*, the proportion coefficient *c* and the random coefficient *r* together determine the proportion of individual experience and group experience in the iteration.

When the iteration of the algorithm meets the set conditions, it generally converges to a certain interval or reaches the maximum number of iterations, the optimization is completed, and the final result is output and retained.

## 3. Solving Process Based on Particle Swarm Optimization Algorithm

Based on the theoretical analysis of zoom lens systems in Section 2.1 and the principles of the PSO algorithm in Section 2.2, this study transforms the problem of determining the initial structure of a zoom lens into an optimization process. The process involves generating candidate solutions for the zoom lens design equations within a defined search domain, then iteratively refining these solutions based on a fitness evaluation function.

**Figure 4:**
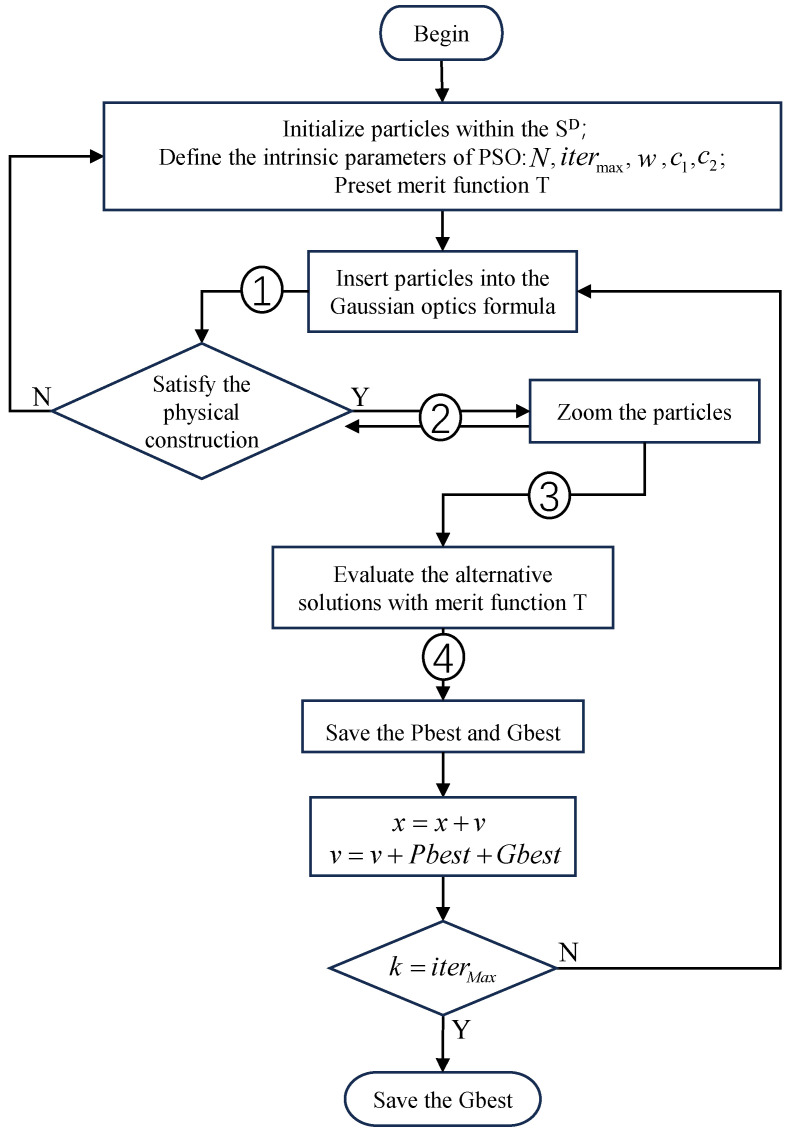
Flowchart of zoom lens initial structure optimization based on particle swarm optimization algorithm.

The process of solving the zoom lens using PSO, as proposed in this paper, is illustrated in Figure 4, and is described in detail below.

The focal powers of the movable lens groups $\varphi_{2}$, $\varphi_{3}$, $\varphi_{4}$, the object distance $l_{2}$ of S2, the spacing $d_{23}$ between S2 and S3, and $d_{34}$ between S3 and S4, are defined as the search space for particles. Particles are randomly initialized within a predefined search domain, ensuring an adequate exploration of the parameter space. The swarm size *N* is specified to ensure the algorithm has appropriate search capabilities within the search space during the iterative process. Additionally, a suitable maximum number of iterations $iter_{max}$ is set to achieve reliable results. The iterative optimization process then begins as follows:

1. Substitute the particles into the Gaussian optical equation $1/l_{i}^{\prime }-1/l_{i}=\varphi_{i}$ and $l_{i+1}=l_{i}^{\prime }-d_{i,i+1}$; then, the image distance $l_{i}^{\prime }$ and magnification $\beta_{i}$ of each lens group can be obtained [9];
2. Check whether each particle satisfies the physical constraints. The defined physical constraints, as shown in Equation (11), are designed to prevent collisions between the moving lens groups during the zooming process and to ensure that the zoom lens can achieve the desired imaging performance,
  (11) $$d_{23}>0.5,d_{34}>0.5,l_{4}^{\prime }>0,\beta_{2}<0,\beta_{3}<0,\beta_{4}>0,b<-2$$
  If the above physical constraints are not satisfied, the particle can not be an alternative solution to the initial structure of the zoom lens, reinitialize it in the search domain.
3. For particles $x_{i}=\left(\varphi_{2},\varphi_{3},\varphi_{4},l_{2},d_{23},d_{34}\right)$ that satisfy physical constraints, a zooming operation is performed as follows: the first three components of the particle remain unchanged, while the last three components are perturbed within their local neighborhoods. The corresponding displacements are then calculated and substituted into the procedures described in Equations (1)–(8) for further computation. If the result of a single zooming operation satisfies the conditions outlined in Equations (1)–(8), a feasible zoom structure for the current focal power is obtained. Since the zoom operation in this paper is based on trial and error, it may not necessarily meet the zoom requirements after a certain displacement of S2 and S4, but this does not mean that the zoom lens cannot be realized under this set of optical power distribution. We set up 1000 trial opportunities to allow the particles to fully explore their adjacent domains under the current optical power, and set an appropriate error range ϵ. We believe that the results under this error can satisfy the subsequent lens design and offer possible solutions to a greater extent. If constraint two still cannot be met after more than 1000 trials, it can be determined that the current configuration no longer satisfy the zoom lens and can be re-initialized in the search domain. Additionally, if more than 20 feasible zoom structures are obtained, the particle and all associated structural information are saved as a candidate solution for the initial design of the zoom lens.
4. After completing the second step, we can obtain the information of an alternative solution. In order to judge the merits of the alternative solution, we construct an evaluation function according to the needs of lens design: we need to achieve the maximum zoom ratio in a limited space, so we need to consider the total length and system zoom ratio; In the initial phase we want to reduce the Petzval field curvature of the system. Because of the need to observe distant objects, we want the system to have a long focal length. Thus, the specific evaluation function *T* is obtained [11,13], as shown in Equation (12), which is a minimization function. The smaller the value, the more the calculation results meet the design requirements,
  (12) $$T=w_{L}\cdot L+w_{R}/R+w_{P}\cdot Petzval+w_{F}/Ft$$
  where *L* is the conjugate length of the zoom part, *R* is the zoom ratio of the lens, Petzval is the Petzval field curve of the lens calculated by $Petzval=1/f_{2}+1/f_{3}+1/f_{4}$, and Ft is the focal length of the telephoto end of the lens calculated by $Ft=f_{1}*m_{max}$. $w_{L}$, $w_{R}$, $w_{P}$, and $w_{F}$ are the coefficient of each item, determine the proportion of each item in the evaluation.
5. After evaluating each alternative solution, record the optimal value of each individual, as well as the group optimal value of the current population and its corresponding particle information, i.e., complete an iteration process.

Before reaching the set maximum number $iter_{max}$ of iterations, the above steps are repeated, and the optimal group history value Gbest and corresponding particle information are obtained in the course of multiple iterations, i.e., the solution of the initial structure of the required zoom lens is completed.

## 4. Verification: Mid-Wave Infrared Zoom Lens Design

Based on the method proposed in this paper, a compact large-zoom-ratio mid-wave infrared system design was completed, with the design specifications outlined in Table 1 below.

In this study, the paraxial optical structure of the designed mid-wave infrared zoom lens is shown in Figure 1. This structure consists of a front fixed group, three moving lens groups, and a relay group. The proposed method was applied to solve for the moving lens group (zoom part), which, in combination with the front fixed group and the secondary imaging group, forms the final optical system.

During the design process, the focal length of the front fixed group was chosen to be 300 mm. As described in Section 2.1, the focal lengths of the lens groups in the zoom part follow a “−+−” configuration. To ensure the feasibility of subsequent design and manufacturing, the specific focal lengths of each lens group were constrained within a reasonable range. Additionally, to prevent collisions between the moving lens groups during zooming, it was necessary to ensure that the spacing between the lens groups remained greater than zero. Furthermore, to explore potential solutions as broadly as possible, the search range for each parameter was moderately expanded. Subsequently, 200 particles were added, and the maximum number of iterations was set to 100 to ensure sufficient global search by the PSO algorithm. After several rounds of simulation analysis and optimization, the coefficients in Equation (12) were set to 0.02, 400, 200, and 3000, respectively.

As shown in Table 2, the parameters were set according to these values. The algorithm was written in MATLAB software (matlab R2017a) following the process described in Figure 4 to calculate the near-axis structure of the mid-wave infrared zoom lens.

**Figure 5:**
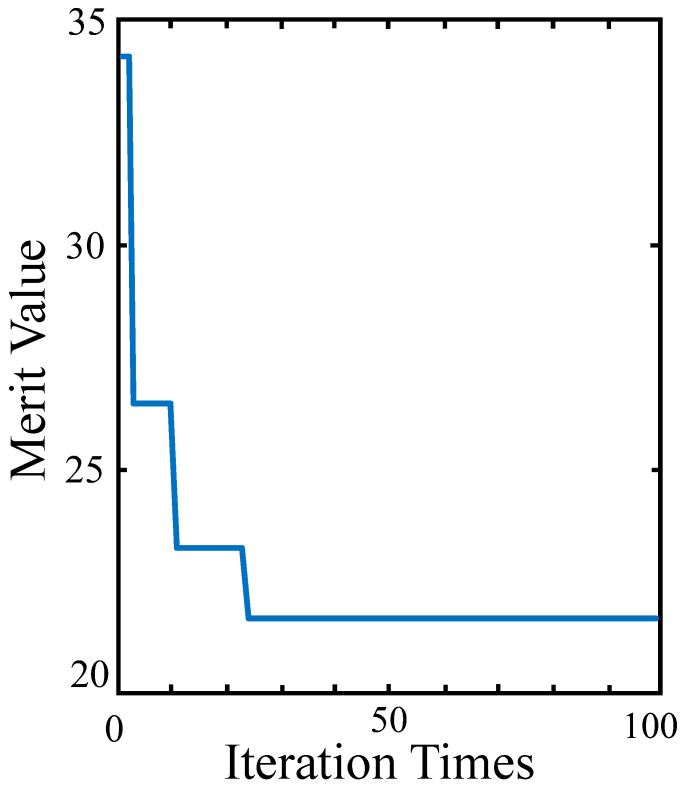
Merit value with respect to the iteration times.

Figure 5 shows the trend of the values of the merit function throughout the entire iteration process. It can be observed that, as the number of iterations increases, the values quickly converge to the global minimum after passing through several local minima.

After the evaluation function converged, we obtain OAL=421.292 mm, R=50.4, Petzval=−0.0183, and $F_{T}=846.129$ mm. The above data do not include the relay group. These data are a satisfactory result obtained by balancing the contribution of various indicators to the evaluation function and slightly increasing the weight for the telephoto focal length. The result provides a good starting point for subsequent design.

The results are partially presented in Table 3 and Table 4. Table 3 shows the focal lengths of each lens group in the zoom part, as well as the zoom ratios of the zoom part when the system is at the telephoto, mid-focus, and wide-angle configurations. Table 3 presents the spacings between each lens group when the system is at the telephoto, mid-focus, and wide-angle configurations.

**Figure 6:**
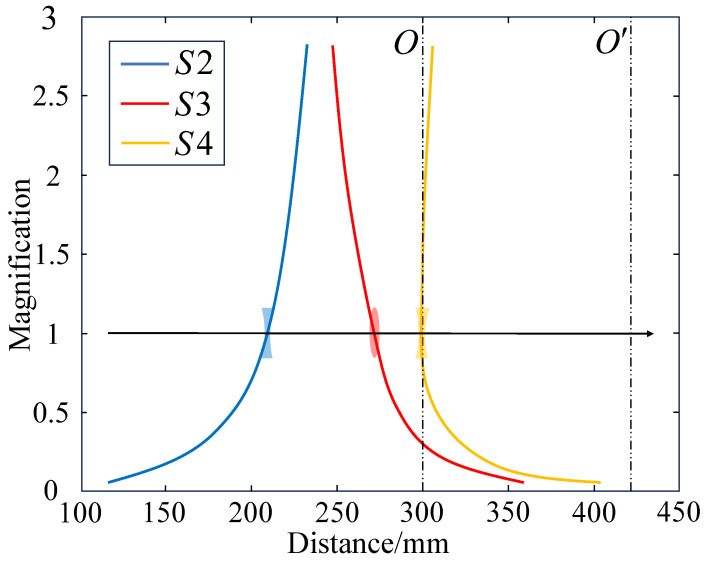
Zoom trajectory of the movable lens group during zooming.

Based on the calculation results, the zoom curve shown in Figure 6 was plotted according to the positions of each lens group at different zoom ratios.

**Figure 7:**
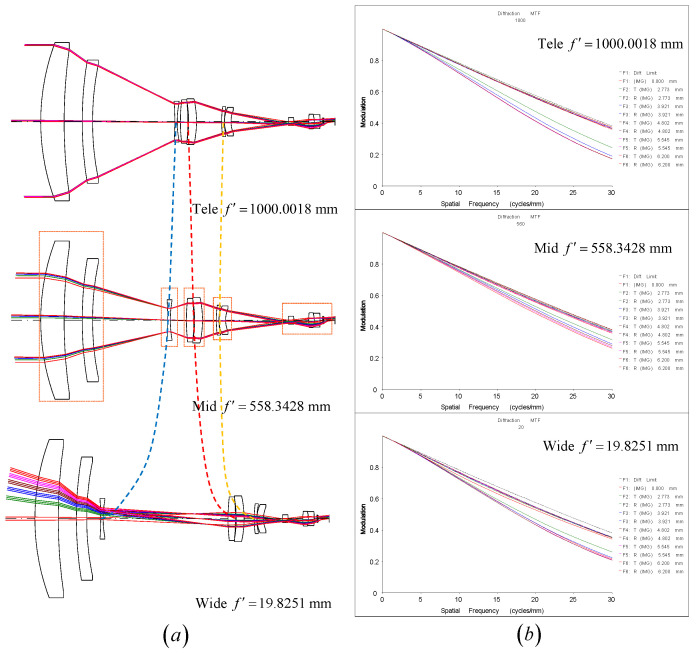
(**a**) Zoom lens at tele-mid-wide constructions. (**b**) MTF of zoom lens at tele-mid-wide constructions.

The results obtained were transformed into a practical lens design using the optical design software CodeV 202303 for aberration correction [17]. The real image height was set to 6.2 mm, and the F-number was set to 4. Additional lens elements were incorporated into each lens group to correct system aberrations: one lens was added to the zoom group S2, while two lenses were added to each of the groups (S1, S3, and S4). Silicon (Si) and germanium (Ge) were used as the lens materials, with ZnS being employed in the relay group for chromatic aberration correction. Aspheric surfaces have been added to correct aberrations. Since real lenses have curvature and thickness, the principal plane positions differ from those of ideal lenses. Thus, during the conversion process, attention was given to the spacings between the principal planes of the lens groups rather than the spacings between lens surfaces. To ensure manufacturability, constraints were applied to the ratio of lens thickness to aperture diameter. Using CodeV’s optimization features, the final zoom lens design and system MTF curve were obtained, as shown in Figure 7. The resulting system lens group focal lengths were $f_{1}=299.9619$ mm, $f_{2}=-35.8408$ mm, $f_{3}=50.0760$ mm, $f_{4}=-97.4869$ mm, $f_{5}=18.8965$ mm, aligning with the calculated values. The total optical length was 514.3636 mm, with a focal length range *F* = (19.8251∼1000.0018 mm), resulting in a zoom ratio of 50.4×. Across the entire focal length range, the system’s MTF at 30 cycles/mm was consistently greater than 0.2. The final design demonstrates that the method proposed in this study can effectively determine the initial structure of a zoom lens, providing a satisfactory starting point for further design.

## 5. Discussion

The proposed method, which integrates particle swarm optimization (PSO) with paraxial optical analysis, demonstrates significant advancements in the design of mid-wavelength infrared (MWIR) zoom lenses. The introduction of a systematic framework addresses key challenges in zoom lens design, including complex parameter interdependencies and the need for a compact system with a high zoom ratio. By leveraging PSO, the optimization process efficiently explored a wide parameter space, producing candidate solutions that meet stringent design criteria.

The incorporation of three movable lens groups instead of the traditional two proved critical in balancing focal power distribution and mitigating aberrations. The iterative refinement through PSO ensured that physical constraints, such as lens group spacing and compensation requirements, were consistently maintained. This approach not only reduced the time and computational effort typically associated with trial-and-error methods, but also resulted in a zoom system with a remarkable zoom ratio of 50× and minimal Petzval field curvature.

Despite these successes, some limitations were observed. The reliance on high computational resources for a broad parameter search and the sensitivity to initial conditions could impact scalability for even larger zoom ratios. Moreover, while the approach effectively generated an initial lens structure, additional manual refinements and aberration corrections using software like CodeV were necessary, suggesting room for integration of advanced aberration correction within the PSO framework.

## 6. Conclusions

This study presents an innovative approach to the design of compact, high-zoom-ratio MWIR zoom lenses by combining differential function of zoom process with particle swarm optimization (PSO). The method addresses critical challenges in lens design, achieving a zoom ratio of 50.4× within a compact configuration while maintaining high optical performance. The systematic framework significantly enhances computational efficiency and design accuracy compared to traditional methods. The successful application to a MWIR system demonstrates the method’s potential for diverse optical applications. Future work could focus on refining the PSO algorithm to incorporate real-time aberration correction and exploring its utility in broader optical design scenarios. This method represents a significant step forward in the field of optical engineering, providing a robust foundation for advanced zoom lens design.

## Figures and Tables

**Table 1:** Design specifications.

Index	Parameter
band (nm)	3700∼4800
Sensor specifications	640 × 512/15 μm
F-Number	4
Focal range (mm)	20∼1000
Max Length (mm)	530

**Table 2:** Parameter settings in PSO algorithm.

Iteration	$S^{D}$	Merit Function
N = 200, iter = 100, w=0.6, $c_{1}$ = 0.8, $c_{2}$ = 1.0	$\varphi_{2}\in$ (−0.1,−0.01), $\varphi_{3}\in$ (0.0125,0.05), $\varphi_{4}\in$ (−0.1,−0.01), $l_{1}\in$ (−300,−100), $d_{12}\in$ (150,250), $d_{23}\in$ (10,200)	$\omega_{L}$ = 0.02, $\omega_{R}$ = 400, $\omega_{P}$ = 200, $\omega_{F}$ = 3000

**Table 3:** Focal length of each group in zoom part and ratio of zoom part at different constructions of 50× zoom lens.

Lens Group	Focal (mm)	Construction	Magnification
S2	−35.490	Wide	0.0559
S3	49.572	Mid	1.5748
S4	−97.285	Tele	2.8204

**Table 4:** Spacings between each group at different constructions.

	$d_{01}$	$d_{12}$	$d_{23}$	$d_{34}$
Wide	114.295	245.261	48.239	13.497
Mid	211.319	56.694	33.513	119.766
Tele	231.115	17.498	57.447	115.232

## Data Availability

Dataset available on request from the authors.
